# The Y chromosome sequence of the channel catfish suggests novel sex determination mechanisms in teleost fish

**DOI:** 10.1186/s12915-019-0627-7

**Published:** 2019-01-25

**Authors:** Lisui Bao, Changxu Tian, Shikai Liu, Yu Zhang, Ahmed Elaswad, Zihao Yuan, Karim Khalil, Fanyue Sun, Yujia Yang, Tao Zhou, Ning Li, Suxu Tan, Qifan Zeng, Yang Liu, Yueru Li, Yun Li, Dongya Gao, Rex Dunham, Kenneth Davis, Geoffrey Waldbieser, Zhanjiang Liu

**Affiliations:** 1 The Fish Molecular Genetics and Biotechnology Laboratory, Aquatic Genomics Unit, School of Fisheries, Aquaculture and Aquatic Sciences, Auburn University, Auburn, AL 36849 USA; 2 USDA-ARS Warmwater Aquaculture Research Unit, P.O. Box 38, 141 Experiment Station Road, Stoneville, MS 38776 USA; 3 Department of Biology, College of Art and Sciences, Syracuse University, Syracuse, NY 13244 USA

**Keywords:** Y chromosome, Sex determination, Catfish, PacBio, RNA-Seq

## Abstract

**Background:**

Sex determination mechanisms in teleost fish broadly differ from mammals and birds, with sex chromosomes that are far less differentiated and recombination often occurring along the length of the X and Y chromosomes, posing major challenges for the identification of specific sex determination genes. Here, we take an innovative approach of comparative genome analysis of the genomic sequences of the X chromosome and newly sequenced Y chromosome in the channel catfish.

**Results:**

Using a YY channel catfish as the sequencing template, we generated, assembled, and annotated the Y genome sequence of channel catfish. The genome sequence assembly had a contig N50 size of 2.7 Mb and a scaffold N50 size of 26.7 Mb. Genetic linkage and GWAS analyses placed the sex determination locus within a genetic distance less than 0.5 cM and physical distance of 8.9 Mb. However, comparison of the channel catfish X and Y chromosome sequences showed no sex-specific genes. Instead, comparative RNA-Seq analysis between females and males revealed exclusive sex-specific expression of an isoform of the breast cancer anti-resistance 1 (BCAR1) gene in the male during early sex differentiation. Experimental knockout of BCAR1 gene converted genetic males (XY) to phenotypic females, suggesting BCAR1 as a putative sex determination gene.

**Conclusions:**

We present the first Y chromosome sequence among teleost fish, and one of the few whole Y chromosome sequences among vertebrate species. Comparative analyses suggest that sex-specific isoform expression through alternative splicing may underlie sex determination processes in the channel catfish, and we identify BCAR1 as a potential sex determination gene.

**Electronic supplementary material:**

The online version of this article (10.1186/s12915-019-0627-7) contains supplementary material, which is available to authorized users.

## Background

The processes of sex determination are tremendously diverse in teleosts and include hermaphroditism and environmental sex determination as well as genetic sex determination [1]. In most cases, the mechanisms of genetic sex determination of teleost fish are quite different from those of tetrapod, even though they originated from the same lineage about 450 million years ago, and share approximately 70% genome sequence similarity [2]. Unlike in mammals and birds, where distinguishable sex chromosomes and common master sex-determining genes are present [3, 4], heterogametic sex chromosomes have only observed in approximately 270 species (less than 1%) of teleost fish; of those, approximately 70% are male heterogametic (XX females and XY males) and 30% are female heterogametic (ZZ males and ZW females) [5–7].

A wide variety of genetic sex-determining mechanisms have been identified in teleost species, with various genes serving as the “master sex-determining genes” such as *DMRT1* in medaka species *Oryzias latipes* and *O. curvinotus* [8, 9], *GSDF* in the medaka species *Oryzias luzonensis* [10], *SDY* in rainbow trout (*Oncorhynchus mykiss*) [11], and *AMHY* in the Patagonian pejerrey (*Odontesthes hatcheri*) [12]. In addition to specific sex-determining genes, a single nucleotide polymorphism (SNP) was reported to be responsible for sex determination in fugu (*Takifugu rubripes*) [13]. Analysis of sex determination patterns in half-smooth tongue sole (*Cynoglossus semilaevis*) revealed that this species has a ZW type sex determination system, and that the dmrt1 gene exhibited characteristics of sex-determining genes such as sex chromosome linkage, male-specific expression, and essentiality for testis development [14], and that its knockout in ZZ fish (male karyotype) led to the development of female phenotypes [15].

In contrast to highly differentiated sex chromosomes in mammals, where the Y chromosome has lost most of its genes compared to the X chromosome, sex chromosomes of fish species are generally less differentiated. The mammalian Y chromosome has been genetically isolated without recombination with the X chromosome beyond the pseudoautosomal region (PAR) [16, 17], but recombination occurs along the length of the catfish Y and X chromosomes. Significant differences between the W and Z chromosomes have been reported in the female heterogametic half-smooth tongue sole where the W chromosome is more than 8 Mb larger than the Z chromosome. However, sex chromosome karyotypes, and presumably sex chromosome lengths, are highly similar in size in most fish species studied to date [17]. Therefore, even though genetic studies can clearly map the sex determination gene to a chromosomal region, identification of sex determination gene is still a daunting task with teleost fish.

In this study, we adopted an innovative approach for the identification of the sex determination gene in channel catfish by comparative genome analysis of the genomic sequences of the X chromosome and the Y chromosome. Through sex reversal, we were able to produce XY phenotypic females, and mating of XY phenotypic females with the normal XY males allowed the production of offsprings carrying XX, XY, or YY sex chromosome sets. The YY fish offered a unique template for the sequencing and assembly of the sex chromosome sequences. Here, we report the generation of a high-quality whole genome assembly of a YY male fish, comparative genome and transcriptome analyses, and identification of the breast cancer anti-resistance 1 (BCAR1) gene as a candidate for the sex-determining locus in channel catfish.

## Results

### Strategy for the identification of the sex determination gene

Resources for the channel catfish genome included a dense genetic map with > 253,000 SNP markers [18] and the reference genome sequence assembly [19]. On average, 1 cM on the genetic map was equivalent to 230 Kb in the reference genome sequence assembly. Because the reference genome was generated using a gynogenetic female, only the X chromosome was represented; therefore, we used hormonal sex reversal- and marker-assisted mating to produce males with two Y chromosomes. A large number of individuals were analyzed to reduce the area of the sex determination locus to a small genomic region which was then located on the X and Y chromosome-specific sequence assemblies.

### Mapping of the sex determination locus

**Fig. 1:**
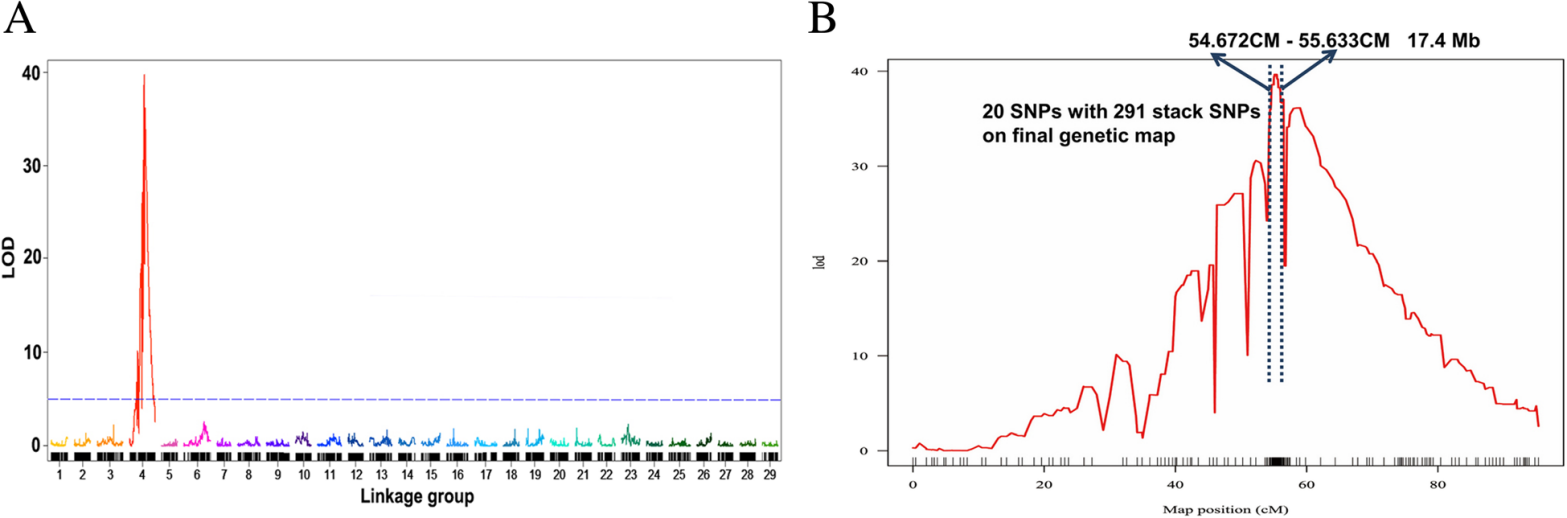
QTL mapping of sex chromosome of channel catfish. **a** The distribution of LOD values of QTL of sex of channel catfish on each linkage group are shown in chromosomal order. **b** The distribution of LOD values of QTL of sex on linkage group (LG) 4 are shown in red line, and 95% Bayes confidence interval is shown within the blue dotted lines

The sex determination locus was located on chromosome 4 (linkage group 4, Fig. 1a) using genetic linkage mapping of 187 fish from one family [20]. This linkage was validated with two more families containing 192 offspring each (data not shown). Detailed linkage analysis indicated that the sex trait was most closely linked with 20 SNP markers spanning a genetic distance of ~ 1.0 cM (Fig. 1b). The LOD score was extremely high (almost 40) with the sex determination locus mapped to chromosome 4. There was no evidence that any other quantitative loci contributed to sex determination (Fig. 1a). However, the genomic location of the linked SNP markers defined a large physical distance of 17.4 Mb on the X chromosome.

Realizing the potential for long haplotype blocks in the F2 offspring, we performed GWAS on unrelated catfish to take advantage of historical recombination which is accumulated over multiple generations within populations to associate traits with individual sequence-level polymorphisms.

**Fig. 2:**
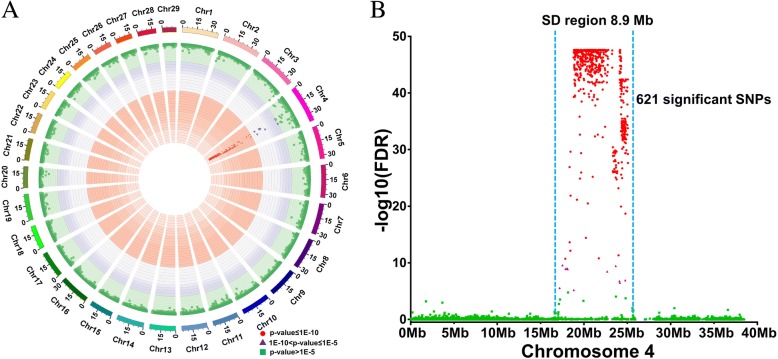
Genome-wide association study (GWAS) of sex determination region of channel catfish. **a** The distribution of significant GWAS SNPs across the whole genome of channel catfish. SNPs with false discovery rate (FDR) *P* value ≤ 1E−10 are shown as red dots. SNPs with FDR 1E−5 > *P* value > 1E−10 are shown as purple triangles. SNPs with FDR *P* value > 1E−5 are shown as green squares. **b** The distribution of significant GWAS SNPs on chromosome 4. The sex determination region is indicated within blue dotted lines

A total of 199 wild channel catfish with sex information, collected from 12 populations in seven watersheds [21], were genotyped with the catfish 250 K SNP arrays [22]. Six hundred and twenty-one SNPs were significantly associated with the sex trait, all on chromosome 4 (Fig. 2a), again pointing to a single locus involved in genetic sex determination of channel catfish. The most significant SNP loci were distributed within a narrower region of 8.9 Mb (Fig. 2b).

### Sequencing and assembly of the YY genome

Channel catfish uses an XY chromosomal sex determination system, and we produced male catfish containing two “Y” chromosomes. Genomic DNA from one YY male was sequenced with Pacific Biosciences long reads to a depth of 57.7X genome coverage (Table 1). These sequences were assembled into 5830 contigs, and 3164 scaffolds, with a contig N50 of 2.7 Mb, and scaffold N50 of 26.7 Mb. The total assembled genome sequence was 1.01 Gb (Table 1). This assembly was significantly longer than the reference catfish genome sequence assembled from Illumina sequencing, because of the inclusion of repetitive sequences in the YY genome sequence assembly.

**Table 1:** Sequencing and assembly of the genome of YY channel catfish

A. Sequences generated for the assembly of YY channel catfish genome				
Libraries	Number of reads	Average read length	Genome coverage	Usage
Raw PacBio reads	5,453,718	10,579 bp	57.7X	Contig assembly and assembly polishing
Corrected PacBio reads	2,240,010	16,192 bp	36.3X	Contig assembly
B. Assembly statistics				
Total number of contigs	Total number of scaffold	Contig N50 (bp)	Scaffold N50 (bp)	Total assembled genome size in scaffolds (bp)
5830	3164	2,695,784	26,683,497	1,010,385,809
C. Assembly assessment				
Complete BUSCOs		Fragmented BUSCOs		Total mapped BUSCOs
2442 (94.4%)		55 (2.1%)		2497 (96.5%)

A total of 96.5% of the channel catfish genes matched the BUSCO set, of which 94.4% were completely matched, and 2.1% partially matched (Table 1c); the YY sequence assembly had a much greater contiguity than the reference genome sequence, as expected for a long sequence assembly, and the gene content also suggested a complete coverage of the YY genome.

### Comparison of sequences and gene contents of X and Y chromosomes

**Fig. 3:**
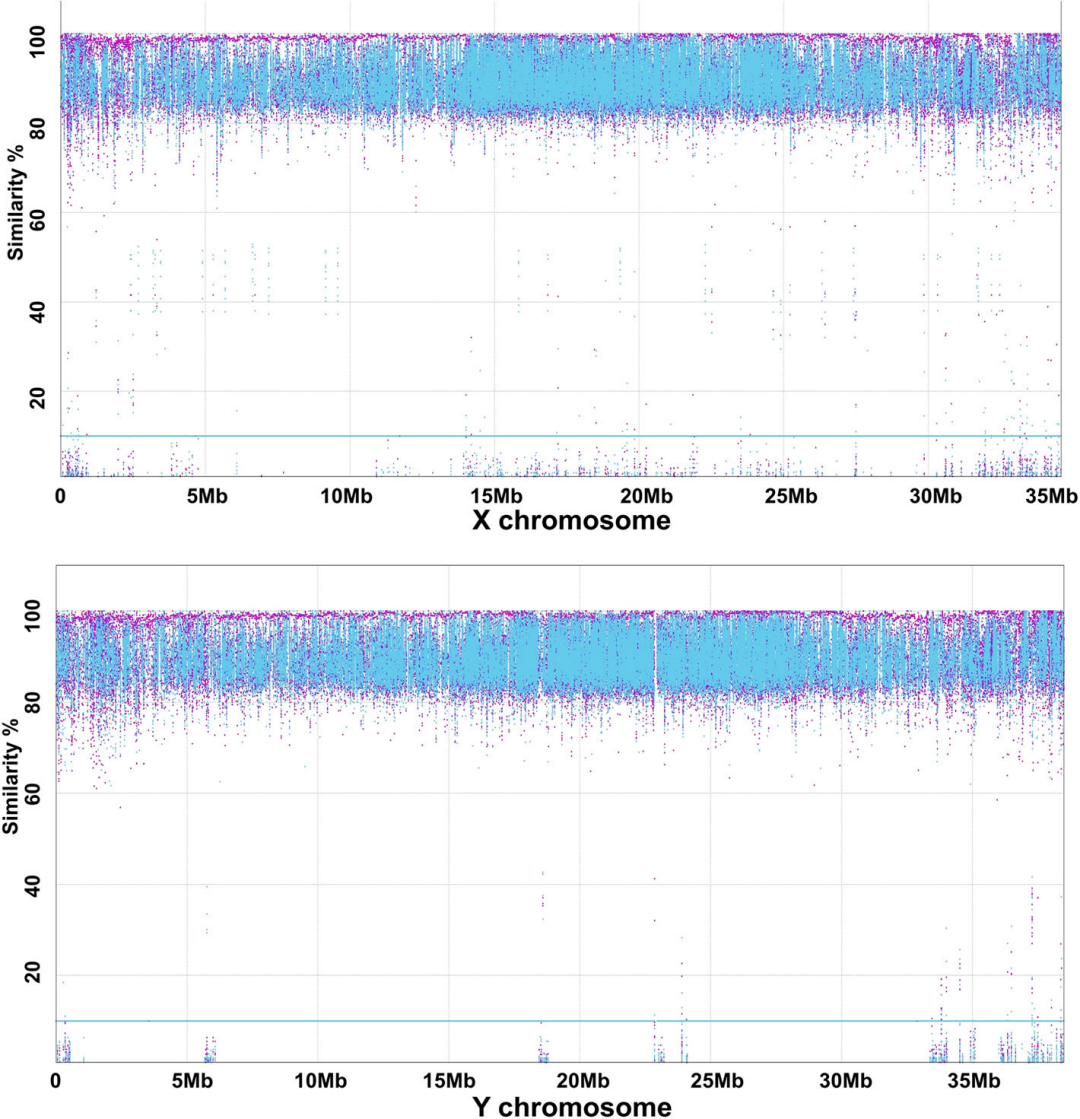
Comparison of sequence similarity of channel catfish X and Y chromosomes. Genomic reads were mapped to chromosome assemblies. *X* axis is the position of the sex chromosome. *Y* axis is the percent similarity. Purple dots indicate the forward read match, and blue dots indicate the reverse read match

We utilized direct sequence comparison and subtraction between the Y and X chromosome assemblies to reveal any sex determination genes. A total of 20 and 39 scaffolds were identified for Y and X chromosome, respectively. These scaffolds represented ~ 38.5 Mb of the Y chromosome sequences, and ~ 34.6 Mb of the X chromosomal sequences (Table 2). We mapped the sequences of the Y chromosome against the X chromosome sequences of the reference genome generated from the XX doubled haploid (Fig. 3a), and vice-versa (Fig. 3b); however, no significant difference in gene sequence was identified from X and Y chromosomes, which is consistent with previous karyotype study of a sex chromosome in many fish species including channel catfish [23, 24].

**Table 2:** Annotation of the channel catfish Y chromosome genes and their comparison with those on the X chromosome

	Y chromosome	X chromosome (Liu et al. 2016)
Number of scaffolds	20	39
Size (bp)	38,493,568	34,595,840
Size without gaps (bp)	38,069,678	34,079,759
Size with repeats masked(bp)	19,977,697	18,310,806
SNPs on genetic map	2122	2270
Genes	950	950
Sex-specific genes	0	0

A total of 950 genes were annotated on the assembled X chromosome, 950 genes were annotated on the Y chromosome, and gene identities were completely shared between the X and Y chromosomes. These results pointed to variation in genic or non-genic sequences in the sex determination region or differential gene expression as a trigger of sex determination.

### Sexually differentially expressed genes during early stages of sex determination

**Fig. 4:**
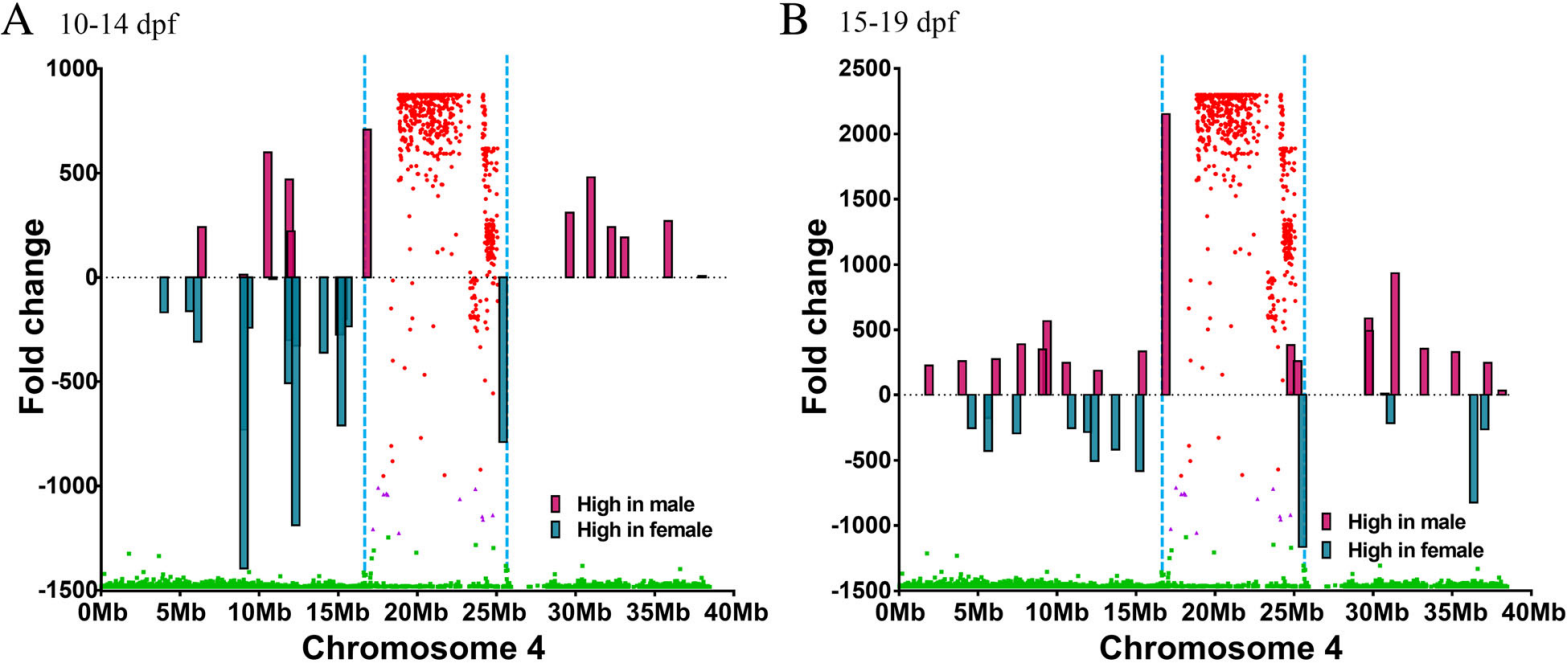
Differentially expressed genes on the channel catfish sex chromosome. Teal bars indicate the genes highly expressed in females, and maroon bars indicate the genes highly expressed in males. The *X* axis shows the position along the sex chromosome, and *Y* axis indicates the fold change in the gene expression. Blue dotted lines indicate the border of the sex determination region. **a** Differentially expressed genes from 10 to 14 days post fertilization (dpf). **b** Differentially expressed genes from 15 to 19 dpf

RNA-Seq was used to detect sexually differentially expressed genes during early stages of sex determination. Although catfish sex is driven by gonadal sex determination, the sex phenotype is not committed until 19 days post fertilization (dpf) [25]. We reasoned that the genes important for sex determination should be differentially expressed in early stages of sex phenotype expression. Therefore, we collected catfish embryos and newly hatched fry daily after fertilization, separated males and females by using a tightly sex-linked marker [26], and then conducted RNA-Seq analysis (Table 3). The short RNA-Seq reads of male and female samples were then mapped to the reference transcriptome assembly, then levels of gene expression were analyzed for sex-specific differences. During 10–14 days post fertilization (dpf), a total of 511 genes were expressed higher in the male than in the female samples, while 754 genes were expressed higher in the female than in the male samples. Similarly, during 15–19 dpf, 800 genes were expressed at high levels in the male samples, while 516 genes were expressed at higher levels in the female samples (Table 4). Differentially expressed genes located on chromosome 4 were reduced to 12 and 20 genes expressed higher in the males 10–14 dpf and 15–19 dpf, respectively, and to 18 and 13 genes expressed higher in the females 10–14 dpf and 15–19 dpf, respectively (Additional file 1: Table S1). Of the 146 genes present within the 8.9 Mb sex determination region, only two were differentially expressed between the males and females at 10–14 dpf: breast cancer anti-estrogen resistance protein 1 (BCAR1) was expressed higher in males and Golgin subfamily B member 1 was expressed higher in females (Fig. 4a). Three genes, A-kinase anchor protein SPHKAP, Spectrin beta chain, and BCAR1, were expressed higher in males at 15–19 dpf while one, a putative leucine-rich repeat-containing protein, was expressed higher in females at this developmental stage (Fig. 4b). Only BCAR1 was differentially expressed in the male in the entire period of 10–19 dpf, and its transcripts were not detected in the females.

**Table 3:** Summary of de novo assembly of catfish transcriptome during sex determination stages using Trinity

Contigs (≥ 200 bp)	469,815
Large contigs (≥ 1000 bp)	94,094
Maximum length (bp)	45,308
Average length (bp)	832.9
N50 (bp)	1718
Reads mapped to final reference (%)	72.04%
Unigene hits	24,442

**Table 4:** Differentially expressed genes between male and female catfish 10–19 days post fertilization (dpf). Fish were sexed by using a microsatellite marker [26]. Expression levels were determined using RNA-Seq datasets

	High/low in male 10–14 dpf	High/low in male 15–19 dpf
Differentially expressed genes across whole genome	511/754	800/516
Differentially expressed genes on LG4	12/18	20/13

**Fig. 5:**
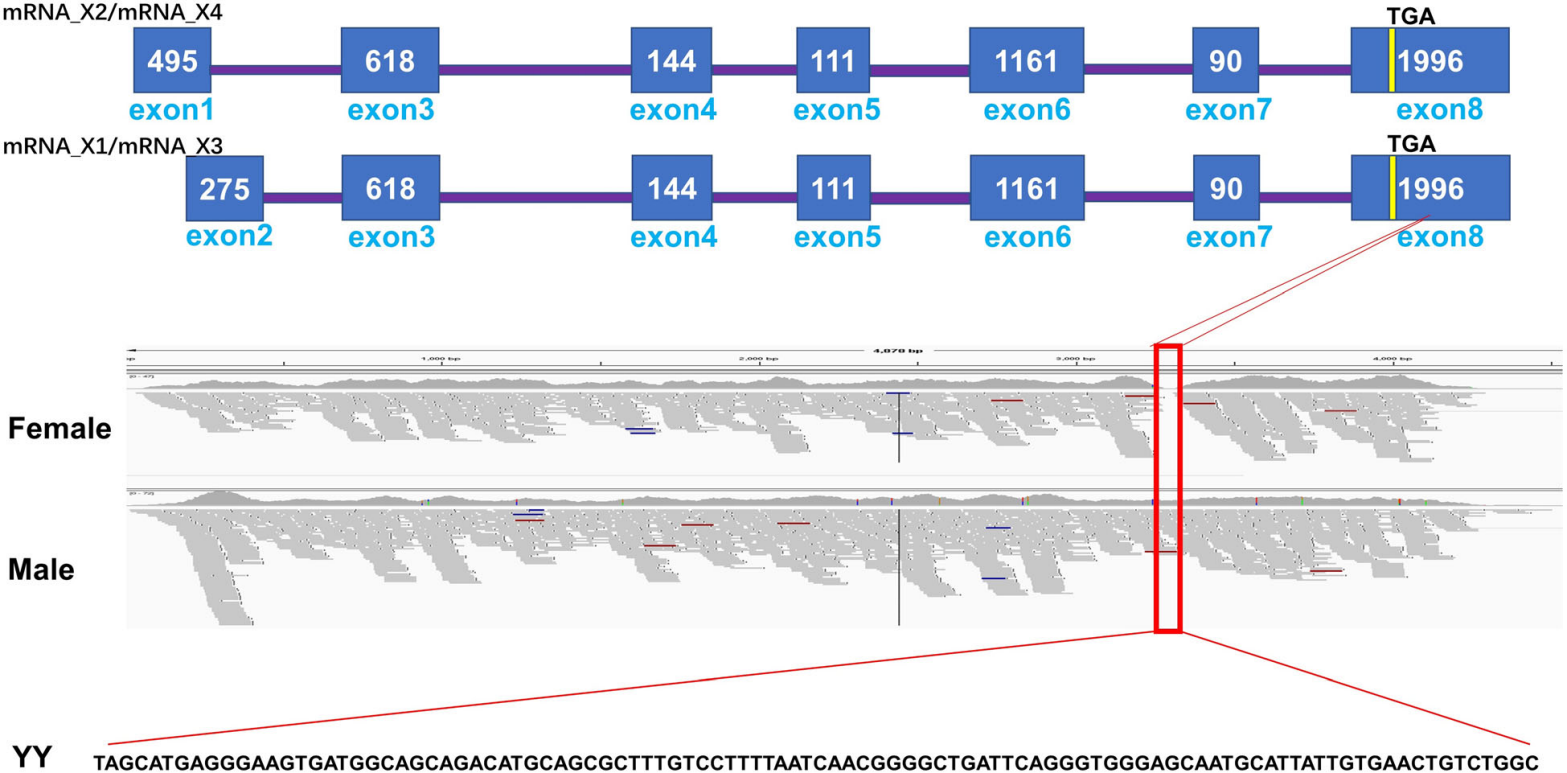
Structure of BCAR1 gene in channel catfish. The BCAR1 genes contain 8 exons, and four transcript isoforms of BCAR1 gene exist. Isoform 2 and isoform 4 start in exon 1 and contain exons 3–8, but have slightly different splicing sites from each other. Isoforms 1 and 3 begin in exon 2 and contain exons 3–8 but slightly different splicing sites. The red lines indicate the position and sequences of the male-specific transcript and that sequence from the YY genome is shown at the bottom

The BCAR1 gene encoded transcripts containing 8 exons and 7 introns and encoding a protein of 882 amino acid (Additional file 2: Figure S1). Detailed analysis of the BCAR1 gene expression revealed four transcript isoforms, but only one was differentially expressed in males but not in females. The isoform transcript contained alternatively spliced 3′-UTR sequence in exon 8 (Fig. 5) present in males but absent in females. There were 246 single base substitutions between the reference X and Y chromosome sequences, but only four were exonic. Two substitutions were neutral, but two led to amino acid substitutions of Glu286Asp and Thr735Ser on the Y and X chromosomes, respectively (Additional file 2: Figure S1). Transcripts produced from the Y chromosome contained 102 bases in exon 8 downstream of the translational stop codon which is spliced from the female transcript. Nineteen potential miRNA targeting sites were identified in the 3′-UTR of BCAR1 (Additional file 3: Table S3). Pathway analysis with differentially expressed genes identified BCAR1 in germ cell-Sertoli cell junction signaling pathway (Additional file 4: Table S4).

### BCAR1 knockout of male genotypes led to female phenotypes

**Fig. 6:**
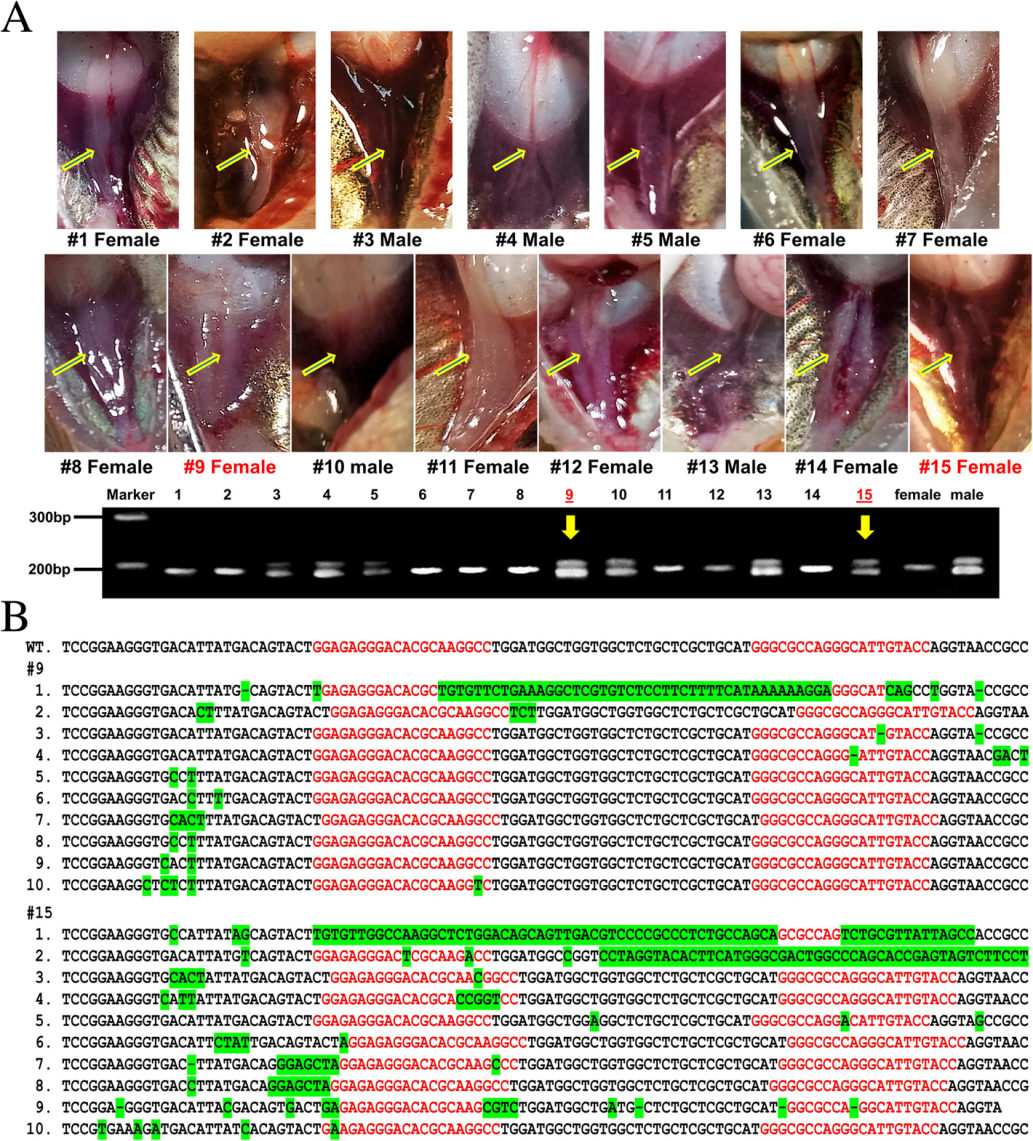
Gonadal tissues from targeted knockout of the BCAR1 gene in channel catfish. **a** Histological examination of gonads 90 days post fertilization from 15 microinjected fish. Arrows indicate the position of the gonad. The agarose gel analysis indicates the genotypic sex of each fish. Males are shown as two bands, and females are shown as one band. Two fish (#9, #15) with male genotypes but female phenotypes are labeled in red. **b** Sequencing analysis of two KO clones. Target sequence is shown in red, mutations and indels are highlighted in green, and short black lines denote deletions WT, wild type

To gain functional support of BCAR1 as a sex determination gene, we knocked out the BCAR1 gene using the CRISPR technology. Fertilized eggs of channel catfish were microinjected with guided RNA and Cas9 mRNA. The resulting embryos were incubated and hatched, and cultured to 90 days after fertilization (approximately 85–86 days post hatching). Sex was determined by microscopic observation of gonadal tissues and by genotyping using sex-specific microsatellite markers. At this developmental stage, female gonadal tissues are well differentiated to two distinct ovarian tubes while the male gonads are thin ribbon-like structures (Fig. 6a). We predicted that genetic knockout of the BCAR1 gene, a candidate sex-determining gene, would lead to a female phenotype in genetic males. Molecular genotyping revealed 7 males and 8 females; however, two of the genotypic males displayed a female phenotype (Fig. 6a). The genomic sequence at the BCAR1 locus of these two fish confirmed disruption of the BCAR1 gene (Fig. 6b).

## Discussion

A great variety of sex determination mechanisms exist in fish among individuals within a population, contrary to the apparent conservation of the gene differentiation network in vertebrates [27]. Generally, the sex determination mechanisms in fish include genetic and environmental regulation. In genetic systems, certain components become dominant in influencing the direction of sex determination [28]. In environmental regulation, the environment in which fish live crucially affects the embryo development. In most cases, this influence is associated with the change of temperature [29]. For example, normally, the sex of channel catfish is determined genetically by the XY system, but female-skewed sex ratios could be generated by applying high temperature during the critical period for sex determination [25]. It is worthwhile to mention that high temperature induce expression of aromatase CYP19A1, a cytochrome that converts testosterone to estradiol, in turn skewing sex ratios toward females. If the same mechanism controls both the sex determination and sex reversal processes in catfish, BCAR1 may interact with estrogen receptor alpha and modulate an estrogen signaling pathway [30]. This observation suggests that the sex of channel catfish is a combination of both genetic sex determination (GSD) and environmental sex determination (ESD), and similar phenomenon has been seen more widespread in fishes than previously believed. In teleost fish, both heterogametic male (XY) and heterogametic female systems exist, with XY system being the major sex determination system. For example, medaka [8], channel catfish [25], and rainbow trout [11] have an XY system. However, many species have female heterogamety (ZW). Fish such as the turbot (*Scophthalmus maximus*) [31], half-smooth tongue sole [32], have a ZW sex determination system.

Much effort has been devoted to analysis of sex determination genes [33–38]. In the genomic era, analysis of sex determination can be accelerated by understanding of sex chromosomes, their structure and organization, and functions [39–41]. However, because of technical difficulties, the whole Y chromosome sequences were generated for only a handful of organisms, all mammals up to date, including human [42], chimpanzee [43], rhesus macaque [44], mouse [45], swine [46], and gorilla [47].

Even though the X and Y chromosomes in mammals are well differentiated, sequencing the Y chromosome in mammalian species is complicated by high levels of repetitive sequence. Teleost X and Y chromosomes are less differentiated and highly similar, and in some cases, sex is determined by a single nucleotide polymorphism [13], making determination of sex chromosome sequences complicated. Sex reversal and production of YY males provided pure sequencing templates of the Y chromosome without homologous X chromosome sequence that could confound assembly [47]. We previously generated a well-assembled XX reference genome from XX gynogen that is a doubled haploid without Y chromosome. The reference genome sequence assembled from this gynogen fish provided the X chromosome sequence. In the current project, we generated whole genome sequence assembly using the YY male fish as the sequencing template. As a result, the sex chromosome sequence assembled from the YY fish are from the Y chromosome without any confusion of the X chromosome sequences. These materials provided a unique system for comparative genome analysis of the X and Y sequences. This innovative approach should be applicable to most, if not all, fish species.

The assembled lengths of sequences from X and Y chromosomes differed by 3.9 Mb (34.59 Mb for X chromosome assembly and 38.49 Mb for the Y chromosome assembly). This difference in length between the X and Y chromosomes as indicated in Table 2 are caused by differences of the sequence assemblies, not by the different lengths of the chromosomes. It must be noted that the X and Y chromosome sequence assemblies were generated by application of different sequencing platforms. The X chromosome sequence was assembled by sequencing a gynogen fish (doubled haploid, no Y chromosome) using mostly the second-generation sequencing platform, Illumina sequencing, supplemented with a low level of PacBio sequencing, while the Y chromosome sequence was assembled by sequencing a YY male fish (no X chromosome) using the third-generation sequencing technology, the PacBio Sequencing. With short Illumina reads, repetitive sequences were not assembled into the scaffolds, leading to the shorter total sequences assembled from X chromosome. With PacBio long reads, the repetitive sequences were mostly read through, and thus, such repetitive sequences were assembled into the final genome assembly, resulted in longer assembled sequences of the Y chromosome. It is the unassembled short repetitive sequences in the sex determination region that made a difference of 3.9 Mb in the final assembly.

Although comparative analysis of sequences generated from different sequencing platforms can be less conclusive, we have the firsthand knowledge of the sequence assemblies of the X and Y chromosomes. In an effort to validate the whole genome sequence assembly, we have developed the 690 K SNP arrays that allowed us to determine the marker positions on the genetic linkage map and on the reference genome sequence assemblies [18]. The collinearity of markers on the linkage map and on the reference genome sequence, and the collinearity of markers on the X and Y chromosomes provided significant confidence of the quality of the assemblies. In addition, to detect sex-specific gene sequences, we took two approaches: First, we conducted a reciprocal mapping of X and Y sequences by alignment of sequences generated from the gynogen and the YY fish after repeat masking. The results (Fig. 3) showed an even coverage across the whole chromosome, with no gaps or leftover gene sequences. Second, we annotated the genes in the assembled X and Y chromosomes, and the gene contents were identical. The unassembled short repetitive sequence pools were also thoroughly analyzed, without detection of any sex-specific sequences. Additionally, the RNA-Seq analysis also did not detect any sex-specific genes.

In this work, we produced a whole genome reference sequence using a YY male as sequencing template, allowing the generation of the first fully assembled Y chromosome sequence outside of mammals, as the very first Y chromosome sequence in teleost fish, and the seventh of all organisms. In addition, channel catfish is the leading aquaculture species in the USA, understanding of its sex determination not only important for research, but also have practical implications for aquaculture. In addition, the Y chromosome sequence should be a valuable resource for studies of sex chromosome biology and evolution.

With a well-assembled female reference genome sequence generated from a XX gynogen [19], and the YY male genome sequence produced in the current research, we were able to combine genome comparative analysis, genetic mapping, and GWAS to narrow the sex determination region to 8.9 Mb. Coupling the positional candidate genes with expression candidate genes during ovary differentiation stage revealed that only a specific transcript of one gene, BCAR1, demonstrated male-specific expression during the phenocritical period (10–19 dpf). Knockout of the BCAR1 gene showed evidence of genetic males with a female phenotype, which provided functional support for BCAR1 as a candidate sex determination gene in channel catfish. What is worth to mention, another transcript, Golgin subfamily B member 1 gene, were found to be expressed highly in female during 10–14 dpf, which means it highly likely participated in ovary differentiation. As it is involved in forming intercisternal cross-bridges of the Golgi complex, the activity of Golgi complex should increase in female during ovary differentiation, which acts to process and package the macromolecules such as proteins and lipids that are synthesized by the cell. Future studies are needed to annotate the function of this gene.

Six master sex-determining genes have been isolated in fish to date: DM-domain gene on the Y chromosome (dmY), the major testis-determining factor in the Japanese medaka (XX-XY) [48], anti-Müllerian hormone (amhY) in the Patagonian pejerrey [12] and anti-Müllerian hormone receptor, type II (amhr2) in fugu [13], gonadal somatic cell-derived factor (gsdf) in the Luzon ricefish (*O. luzonensis*) [49], sexually dimorphic on the Y chromosome gene (sdY) in the rainbow trout [11], and finally SRY-related HMG-Box gene 3 (sox3) in the medaka (*Oryzias dancena*) [50]. Until 2011, all four vertebrate master SD genes were known to code for transcription factors which could have been construed as evidence that gonadal sex determination in vertebrates is always triggered by transcription factors. However, the three novel candidates for the master SD genes in the Patagonian pejerrey, *Oryzias luzonensis*, and fugu code for growth factors or one of their receptors. Thus, these findings suggest alternative mechanisms of genotypic sex determination, in which the main trigger is not constrained to be a transcription factor [37, 51].

In many non-mammalian vertebrate species of fish, newts, frogs, and turtles, exposure to estrogen during early gonadal development can override genetic sex and cause male-to-female sex reversal [52–57]. It is proposed that estrogen acts in the native pathway by which the bipotential gonad forms an ovary in these species [58]. These results suggest that endogenous estrogen feminizes the medulla of the bipotential turtle gonad by inhibiting SOX9 expression [59].

**Fig. 7:**
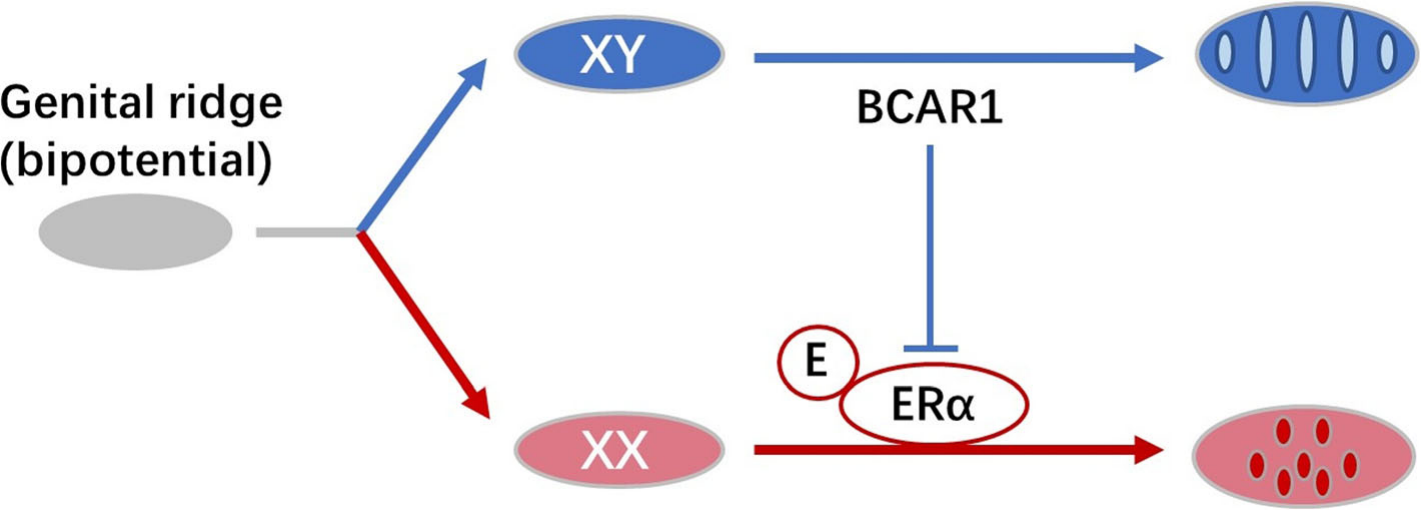
One hypothetical mechanism of BCAR1 involvement in sex determination. The Y-linked BCAR1 isoform inhibits estrogen/estrogen receptor alpha-mediated signal transduction in the undifferentiated gonad and drives development toward the testis

While molecular mechanisms for BCAR1’s function as a sex determination gene are unknown at present, it may function through the control of estrogen/estrogen receptor-mediated signal transduction (Fig. 7). The adaptor protein BCAR1 can be present in a multimolecular complex with estrogen receptor alpha (ERα) [30, 60], and it is known that estrogen downregulates Sox9 levels. In the absence of Sox9, the mammalian embryo undergoes development and differentiation of ovarian tissue. Exposure to exogenous estrogen can lead to sex reversal to females.

The two amino acid changes observed between the X and Y haplotypes of BCAR1 are conservative, one contains the acidic glutamic acid or aspartic acid, the other contains the hydroxylic threonine or serine. The Thr735Ser is included in the Crk-associated substrate domain of BCAR1which is crucial for protein interaction of estrogen receptor. One hypothesis is that this substitution alters the interaction of BCAR1 with the estrogen receptor. It is reported that in breast cancer cells, overexpression of BCAR1 increases c-Src kinase activity and modulates non-genomic estrogen signaling [30]. Interestingly, BCAR1 was identified in germ cell-Sertoli cell junction signaling pathway using pathway analysis with differentially expressed genes during ovary differentiation (Additional file 5: Figure S2). Sertoli cells are supporting cells within the developing testis, and the germ cell-Sertoli cell junction signaling is regulated by testosterone and TGF-β signaling pathway. Testosterone could be converted into estrogen by P450 aromatase; in channel catfish, such a conversion was observed to be almost always the case because both estrogens and testosterone led to sex reversal into females. TGF-β family members such as amh and gsdf are also of great importance in sex determination and gonadal somatic cell development. It is well known that sex determination gene initiates the differentiation of the supporting cells in the indifferent gonad to Sertoli cells, which subsequently form tight and adherent junctions to establish the testis cord [61]. Taken all together, it is possible that the activation of BCAR1 in XY cells interacts with estrogen receptor-α and suppresses estrogen-based ovarian developmental signaling and drives the bipotential genital ridge toward testis (Fig. 7). Future research is warranted to test this hypothesis and to investigate the mechanisms of how BCAR1 may function as a sex determination gene.

Alternately, sex determination in catfish can be regulated at the RNA level, and the additional 102 bp in the male-specific transcript that is spliced in females contains 3 potential miRNA targeting sites. Future studies are warranted to determine the mechanisms of alternative splicing and its involvement in sex determination in catfish. The importance of miRNA target sites suggested that the alternatively spliced RNA may have a different fate upon interactions with miRNA, but these works are beyond the scope of this paper.

## Conclusions

We sequenced and assembled the Y chromosome of channel catfish with a unique strategy of creating a male with two Y chromosomes (normal males are XY), thus avoiding any X chromosome sequences in the assembly. This is the first Y chromosome sequence outside of only a handful of mammalian Y chromosome sequences. We identified a candidate gene for sex determination in catfish through positional and functional evidence. Because BCAR1 was both a positional and expression candidate, and experimental knockout of the BCAR1 gene converted genetic males (XY) to phenotypic females, we posit BCAR1 as a candidate locus for sex determination in channel catfish.

## Methods

### Fish sources and sampling for whole genome sequencing

All procedures involving the handling and treatment of used fish during this study was approved by the Auburn University Institutional Animal Care and Use Committee (AU-IACUC) prior to initiation of the project. All animal procedures were carried out according to the Guide for the Care and Use of Laboratory Animals and the Animal Welfare Act in the USA. The wild channel catfish used in this project were obtained from 12 populations of 7 major watersheds in Alabama [21]. The YY catfish genome donor was derived from the USDA103 catfish strain [61].

### Generation of the YY male channel catfish genome donor

**Fig. 8:**
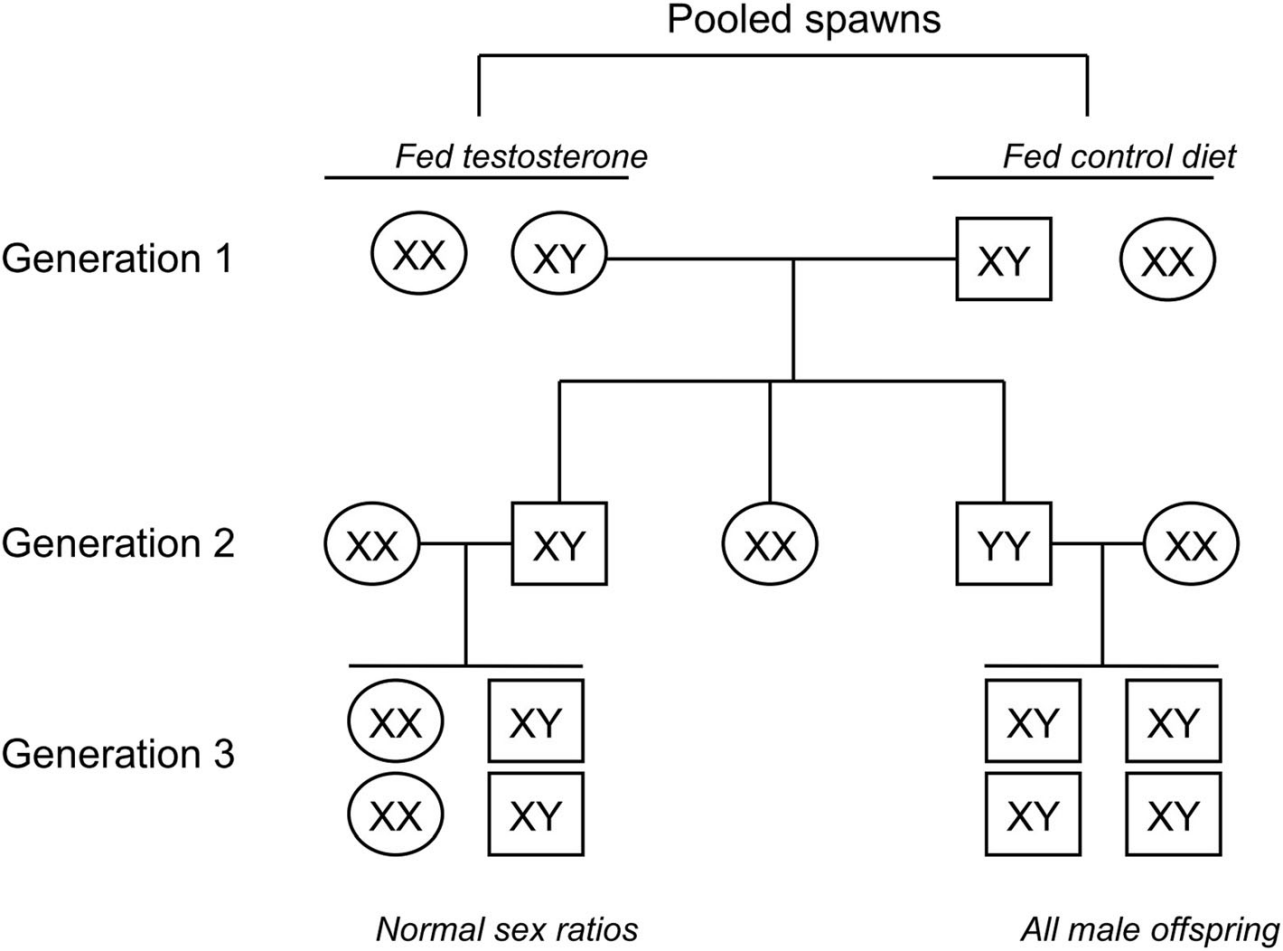
Generation of YY catfish. Channel catfish were fed testosterone to produce generation 1 sex-reversed XY females which were then mated with normal XY males. Generation 2 YY males were identified using molecular markers, and YY genotype was validated through production of all male offspring in generation 3

Three spawns were collected from ponds and hatched in flow-through water, and each family was split into two hatchery tanks. The fry in the sex-reversed treatment were fed ad libitum a 45% crude protein ration containing 60 μg of 17α-Methyltestosterone (Sigma-Aldrich Corp., St. Louis, MO) per gram feed beginning 6 days post hatch [25, 62] while control fry were fed the same ration without testosterone. After 3 weeks of feeding, the fry were raised under normal culture conditions. Channel catfish phenotypic sex is governed by an XY sex-chromosome system with heterogametic males. At 2 and 3 years of age, the treated females were mated with normal XY males in ponds. Spawn parentage was performed using microsatellite markers [63] and also sex-linked microsatellite loci. Males with a YY sex genotype were identified by alternate haplotypes of XX females. At 2 years of age, the putative YY males were mated with normal, unrelated XX females and spawns with all male progeny validated the YY genotype of the sires (Fig. 8).

### Experimental animals and tissue collection for RNA-Seq

Three-year-old channel catfish that displayed physical characteristics of sexual maturity were harvested from ponds (E.W. Shell Fisheries Research Center, Auburn University, Alabama, USA) and held in tanks. The general artificial spawning, fertilization, and husbandry procedures were performed as previously described [64, 65]. In brief, females were injected with luteinizing hormone-releasing hormone analog (LHRHa) at 100 μg/kg body weight, and then, eggs were hand-stripped and fertilized artificially. After hatching, fry were reared in a trough (304 cm × 60 cm × 30 cm) with daily water quality monitoring to maintain a constant water flow of 8–10 L/min, temperature at 28 °C, and dissolved oxygen at 6 ppm. Fry were fed daily to satiation with the Aquamax Fry Starter100 (Cat#: 000-5553, Aquamax, MO, USA). Approximately 50 fry were collected each day starting from the 10th day post fertilization (dpf) to 19th dpf. Fry were euthanized with tricaine methanesulfonate (MS 222) at 300 mg/L before sample collection. Each fry was cut into two portions of head and body. The samples were flash-frozen and stored at − 80 °C until DNA extraction and RNA extraction.

### Sex genotyping

Thirty catfish fry samples were selected each day for sex-linked genotyping using the microsatellite marker AUEST0678 [26]. Genomic DNA was extracted from the head sample of each catfish fry using the Gentra Puregene Tissue Kit (QIAGEN, USA), following manufacturer’s instructions. A touchdown PCR profile was performed as previously described [26], and amplification products were analyzed on a 7% polyacrylamide gel using a LICOR 4300 DNA Analyzer (LICOR Biosciences, Lincoln, NE). After gel electrophoresis, the sex of a catfish fry was determined based on the specific banding pattern that is one shared band (~ 212 bp) was observed in both males and females, while a 205-bp product was present only in male channel catfish [26].

### DNA extraction, library preparation, and PacBio sequencing

Peripheral blood was collected from a YY male catfish and stored at − 80 °C until DNA extraction. To obtain high molecular weight genomic DNA, 15-μl whole blood was digested in a 500-μl solution containing 10-mM Tris-Cl [pH 8.0], 25-mM EDTA, 100-mM NaCl, 0.5% SDS, and 100-μg/ml proteinase K overnight at room temperature. Proteins were precipitated by addition of ½ volume 7.5 M ammonium acetate, gentle mixing for 10 min, and centrifugation for 10 min at 18,000 × *g*, 4 °C. The supernatant was decanted into a 2-ml microcentrifuge tube, and nucleic acids were precipitated by addition of 0.6 volumes isopropanol. The nucleic acids were pelleted by brief centrifugation, the supernatant was removed, and the pellet was dissolved in 400-μl TE buffer. Carbohydrates were removed by addition of 0.3 volumes ethanol, gentle mixing for 10 min, and then centrifugation for 10 min at 18,000 × *g*. The supernatant was decanted to a new microcentrifuge tube, and nucleic acids were re-precipitated with 1.5 volumes of ethanol, washed in 70% ethanol, and resuspended in TE buffer. The purified genomic DNA (A260/280 > 1.8, A260/230 > 2.0) was verified as high molecular weight via agarose gel electrophoresis. Long read sequencing was performed by Pacific Biosciences (Menlo Park, CA, USA) with two Sequel SMRT Cell 1 m v2 Trays on the Sequel platform and by the USDA, ARS, Genomics and Bioinformatics Research Unit (Stoneville, MS) with 20 SMRT cells on the Pacific Biosciences RS II platform.

### De novo assembly of the YY genome

The bam files from the Sequel system were first converted to FASTQ format and assembled with sequences generated from PacBio RS II system. Both Canu [66] and Miniasm [67] were used in parallel to generate the assembly of YY genome of channel catfish. The best assembly was evaluated according to the number of contigs generated, N50 size, average contig size, and maximum contig length. Racon [68] was used to further omit the sequencing errors and call high-quality consensus for the Miniasm assembly. Quickmerge was used to merge the Canu and Miniasm assemblies to further improve contiguity and completeness. Finally, Arrow (Pacific Biosciences), an upgrade from Quiver [69], was used to polish the consensus sequences and further improve the accuracy of the assembly.

The YY genome scaffolds were produced using Chromosomer [70] to align and scaffold YY contigs against the XX reference genome [19]. Where appropriate, estimated gap distances were transferred from the XX reference genome as ambiguous nucleotides (Ns). Chromosome level scaffolding and further correction was conducted by integrating the YY genome assembly with the high-density SNP-based genetic map [20]. The final genetic linkage map was comprised of 54,342 markers (at 29,081 unique positions) distributed across a genetic distance of 3505.4 cM. The 70 bp of sequence flanking each SNP locus on the genetic map was mapped to the assembly scaffolds by BLASTN alignment (*E* value maximal threshold 1e−10, > 95% sequence similarity, > 65-bp alignment length). Scaffolds that were adjacent on a chromosome were manually joined with a string of 100 “*N*s” to represent the gaps between the two adjacent scaffolds based on the high-resolution genetic map.

The assembly quality was assessed by comparison with the benchmarking universal single-copy orthologs (BUSCO; [71]) with the “vertebrate” data set and with the channel catfish reference genome.

### QTL mapping and GWAS of the sex determination region

For QTL mapping of the sex determination (SD) locus, we first constructed a genetic map by genotyping 187 fish from a single family using the catfish 250 K SNP array [22]. The sex phenotype of each fish was determined by examination of reproductive tissues after anesthetization and recorded as a binary trait. Next, 3418 informative SNPs on the genetic map were selected based on the pedigree information and quality value. Linkage between each marker and the SD locus was estimated using the “scanone” function with EM algorithm and binary model integrated in R/qtl software [72]. Significant LOD threshold was obtained by permutation tests with 1000 replicates. The 95% Bayes confidence interval was determined using the “bayesint” function.

For GWAS analysis of the SD locus, 199 wild catfish were genotyped using the catfish 250 K SNP array and their genders were determined using sex-linked markers. The analysis was performed with a linear regression model using SNP & Variation Suite 7 (Golden Helix, Inc., Bozeman, MT). Samples passing the quality control (dish value > 0.85), and SNP call rate threshold (> 95%) were retained for analysis. SNPs with missing genotypes > 5% and minor allele frequency < 5% were removed. The Manhattan plot of significant SNPs was generated by Circos [73].

### Annotation of the YY genome assembly

A library of catfish-specific repetitive sequences was first produced using RepeatModeler v1.0.8 (http://www.repeatmasker.org/RepeatModeler/). Next, the repeat-annotated library was used to mask the genome assembly using RepeatMasker with the option of -nolow and -s. Finally, Augustus [74] and Fgenesh (http://www.softberry.com) were used to predict genes within the repeat-masked assembly. Gene model parameters for Augustus were trained from conserved genes from vertebrate species using CEGMA [75]. The amino acids predicted from the same genomic positon shared by both algorithms were retained and integrated for the downstream homolog-based annotation. Then, the amino acid sequences of the predicted genes were retrieved and queried against the Uniprot and NCBI non-redundant (NR) databases using BLASTP to identify the homologous genes with an *E* value maximum threshold of 1e−5. The names of predicted channel catfish genes were assigned based on their homologous proteins. All of the genes with names of retrotransposable element, transposon, reverse transcriptase, RNA-directed DNA polymerase from transposon, transposase, and transposable elements were treated as potential repetitive elements and removed from the gene annotation. All of the genes with names of uncharacterized, unnamed, unknown, hypothetical, and predicted protein were treated as unnamed genes and searched against Uniprot and NR database as above to return the top 10 matches. Any matches with names of potential repetitive elements were excluded from the annotation. The chromosomal position of each gene was assigned based on the chromosome level assembly, and genes with adjacent locations and identical gene names were labeled. Then, the tandem duplicated genes among the labeled genes were identified using MCScanX [76], and the remaining genes were treated as fragments of a same gene and subgrouped separately based on their position and names. Only the longest protein of each subgroup was kept representing the corresponding gene.

### Comparative analysis of X and Y chromosome sequences

In order to identify the Y-specific fragments, the X chromosome from the reference assembly and the Y chromosome from the present assembly were aligned using MUMmer [77]. Also, the male and female genome assemblies were compared using BLASTN (*E* value < 1e−10, and identity > 95%. MUMmer was also used to perform the genome-wide alignment.

### High throughput RNA sequencing

A total of 10 female and 10 male fry with conclusive determination of sexes were randomly selected from each day for RNA-Seq sequencing. The tissues of 10 male catfish fry collected in the same day were pooled and homogenized with mortar and pestle in the presence of liquid nitrogen. Similarly, the tissues of 10 female catfish fry that were collected in the same day were also pooled and homogenized. Total RNA was extracted using the RNeasy Kit (Qiagen, Valencia, CA, USA) according to the manufacturer’s instructions. RNA concentration and integrity were measured on an Agilent 2100 Bioanalyzer. Equal amounts of RNA from each 5-day period were pooled from 10 to 14 dpf and 15 to 19 dpf for male and female, respectively. These four pooled samples were outsourced for RNA-Seq at the HudsonAlpha Genomic Services Lab (Huntsville, AL, USA). The RNA-Seq libraries were prepared following the standard TruSeq protocols and were sequenced on an Illumina HiSeq 2500 instrument for 100 bp paired-end reads.

### De novo transcriptome assembly and annotation

The de novo assembly of short reads was performed using Trinity (2012-10-05 release) with RNA-Seq reads sequenced from all eight samples. Before assembly, raw reads were trimmed by removing adaptor sequences and ambiguous nucleotides. Reads with quality scores < 20 and length < 30 bp were filtered, and the resulting high-quality sequences were used in the subsequent assembly. The short reads were assembled into unique transcripts using “Inchworm” with a greedy k-mer extension (k-mer = 25). After mapping reads to Inchworm-assembled contigs, “Chrysalis” incorporated reads into deBruijn graphs and the “Butterfly” module processed the individual graphs to generate full-length transcripts. In order to reduce redundancy, the assembly was passed to the CD-Hit (version 4.5.4) for multiple alignments [78]. The threshold of sequence identity was set at 1.0, with the alignment covering > 90% of the shorter sequence. The assembled transcript contigs were annotated by searching against the NCBI zebrafish RefSeq protein database, the UniProtKB/SwissProt database, and the non-redundant (nr) protein database using the BLASTX program with *E* value ≤ 1e−5.

### Identification of differentially expressed genes

High-quality reads from each sample were mapped onto the assembled transcript sequences using Bowtie software [79] with default parameters. The RSEM program [80] was then used to estimate the expression abundance of the transcripts. The total number of mapped reads for each transcript was determined and then normalized to determine FPKM (fragments per kilobase of transcript per million mapped reads). Differential expression analysis was performed using the “edgeR” package [81]. All the samples were normalized by the trimmed mean of *M* values (TMM) [82] which equated overall gene expression levels between samples under the assumption that the majority were not differentially expressed. Transcripts with absolute fold change values of greater than 1.5 and a false discovery rate-adjusted *P* value < 0.05 were included in the analysis as differentially expressed genes. Ingenuity Pathway Analysis (IPA) approaches were used to analyze the differential expression data.

### Identification of miRNA in 3′-UTR of BCAR1

The sequences of miRNA of channel catfish were retrieved from previous study [83]. Potential miRNA targeting sites on 3′-UTR of BCAR1 were identified using RNA22 v2 microRNA target detection [84] with minimum number of 15 paired-up bases in heteroduplex and maximum folding energy as − 15 for heteroduplex (Kcal/mol).

### CRISPR/Cas9 microinjection

Single guide RNA (sgRNA) sequences targeting the BCAR1 gene were searched against the whole genome and any other hits with identity larger than 95% and alignment length larger than 16 bp were labeled as potential off-targets. SgRNA with 0 off-targets were selected, designed and obtained from Transposagen (Lexington, KY), and microinjected together with the Cas9 nuclease mRNA (target sequence1 5′-GGAGAGGGACACGCAAGGCC-3′; target sequence2 5′-GGGCGCCAGGGCATTGTACC-3′). Channel catfish eggs were manually stripped from female broodstock, fertilized with normal sperm, and incubated in fresh water for 30 min. One hundred eggs were placed on a 10-ml Petri dish and 100 pg sgRNA, and 1 ng Cas9 mRNA were injected into the blastodisc using an Eppendorf Microinjector 5242 system (Hamburg, Germany). The control group was microinjected with the same solution without mRNAs. Then, embryos were moved into 10-L tubs filled with Holfreter’s solution containing 10 ppm doxycycline and incubated until hatch. Dead embryos were removed, and water was changed daily. Channel catfish fry were then transferred into a recirculating system.

### TA clone and sequencing

To identify specific modifications of the BCAR1 gene after the knockout experiment, primers were designed flanking the target sites and PCR products were amplified from each individual using the IsoPure DNA Purification Kit (Denville Scientific, Holliston, MA, USA). Purified PCR products were cloned into the TOPO TA Cloning Kit for Sequencing (Invitrogen, Grand Island, NY), and ten transformed recombinant colonies per individual fish were selected for Sanger sequencing (forward primer: 5′-TGGCCAAGGCTCTGTATGAC-3′; reverse primer 5′-CACTTGTGGGTGGTACCTGG-3′) (Eurofins Genomics, Louisville, KY).

## Additional files

Additional file 1: **Table S1.** Differentially expressed genes located on chromosome 4 10-14 dpf. **Table S2.** Differentially expressed genes located on chromosome 4 15-19 dpf. (XLSX 26 kb)

Additional file 2: **Figure S1.** Amino acid comparison of BCAR1 gene between XX and YY catfish. (JPG 1401 kb)

Additional file 3: **Table S3.** Potential miRNA targeting site in 3′-UTR of BCAR1. (DOCX 20 kb)

Additional file 4: **Table S4.** Differentially expressed genes in germ cell-Sertoli cell junction signaling pathway. (DOCX 14 kb)

Additional file 5: **Figure S2.** Germ cell-Sertoli cell junction signaling pathway. (JPG 144 kb)
