# Supplementary figures and images for: The Y chromosome sequence of the channel catfish suggests novel sex determination mechanisms in teleost fish
Source: BMC Biol. 2019 Jan 25;17:6. doi: 10.1186/s12915-019-0627-7 (PMC6346536; doi:10.1186/s12915-019-0627-7)

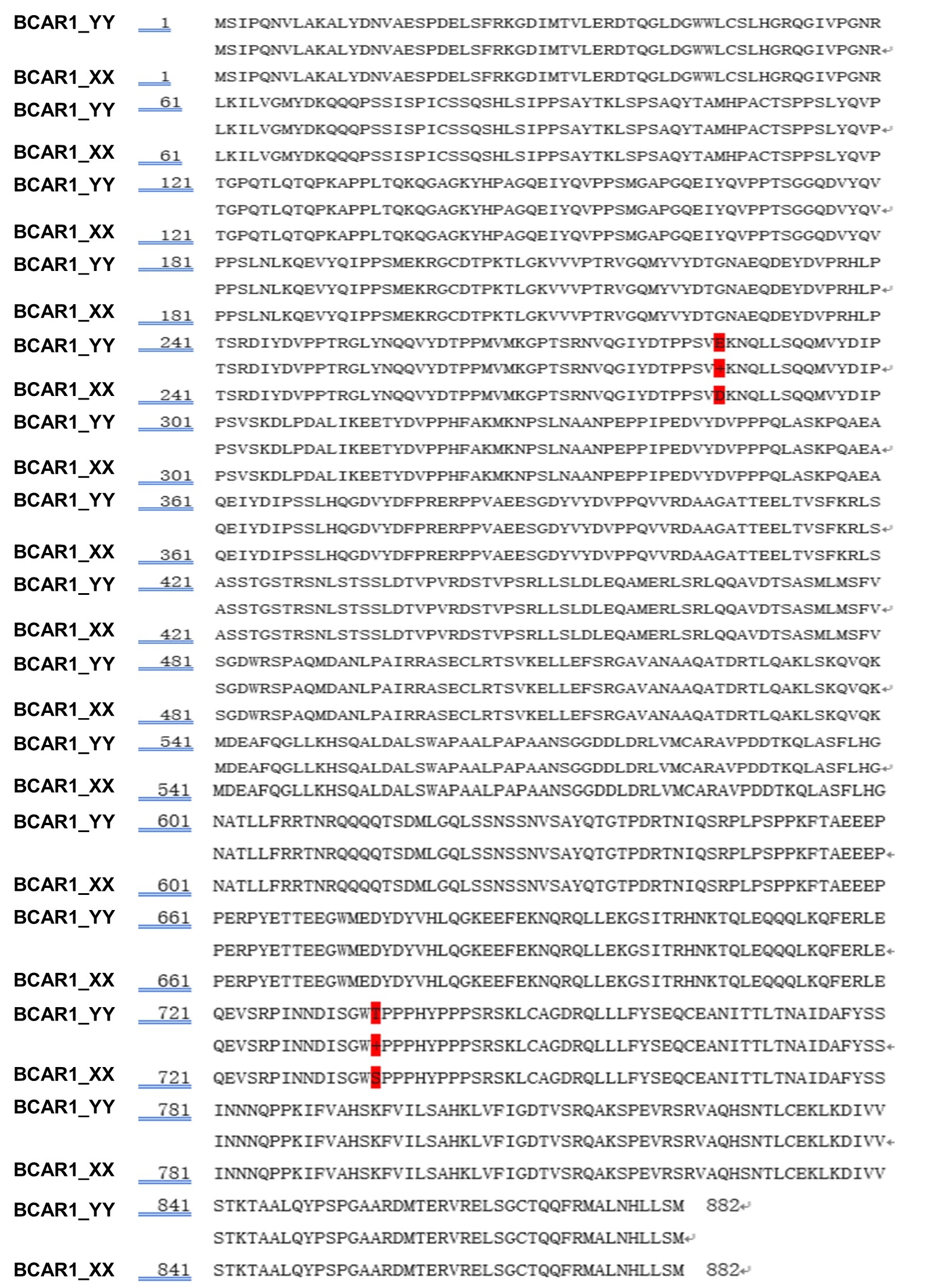

Supplement: Supplementary file 2 — Figure S1. Amino acid comparison of BCAR1 gene between XX and YY catfish. (JPG 1401 kb) [file 12915_2019_627_MOESM2_ESM.jpg]

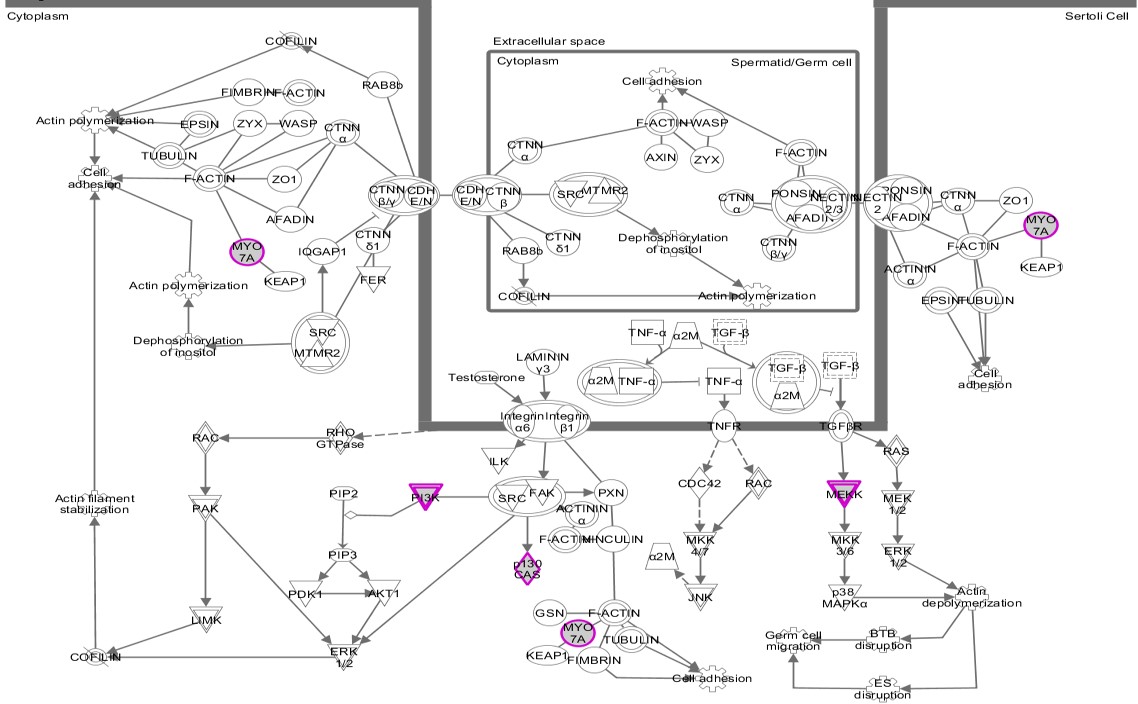

Supplement: Supplementary file 5 — Figure S2. Germ cell-Sertoli cell junction signaling pathway. (JPG 144 kb) [file 12915_2019_627_MOESM5_ESM.jpg]
