# Supplementary material for: The Y chromosome sequence of the channel catfish suggests novel sex determination mechanisms in teleost fish
Source: BMC Biol. 2019 Jan 25;17:6. doi: 10.1186/s12915-019-0627-7 (PMC6346536; doi:10.1186/s12915-019-0627-7)
Supplement: Supplementary file 3 — Table S3. Potential miRNA targeting site in 3′-UTR of BCAR1. (DOCX 20 kb) [file 12915_2019_627_MOESM3_ESM.docx]

**Table S3** Potential miRNA targeting site in 3’ UTR of BCAR1. Positions in male-specific transcript are highlighted.

| miRNA | transcript | Predicted target site | Folding energy (-Kal/mol) | Heteroduplex | P value |
| --- | --- | --- | --- | --- | --- |
| ipu-miR-27a | BCAR1_3UTR | 537 | -17.40 | GGTGGGAGCAATGCATTATTGTGAA :\|:\|\| \|:\| \|\| :\|:\|\|\|\|\|\| TCGCC-TTGAATCG--GTGACACTT | 2.82E-1 |
| ipu-miR-27d | BCAR1_3UTR | 538 | -16.50 | GTGG-GAGCAATGCATTATTGTGAA \|\|\|: \|\|:\| \|\| :\|:\|\|\|\|\|\| CACTTCTTGAATCG--GTGACACTT | 2.82E-1 |
| ipu-miR-92a-1 | BCAR1_3UTR | 533 | -18.10 | TCAGGGTGGGAGCAA-TGCATTA  \|\|\| :\|\|\|\| \|\|\| \|\|\|\| \|\| TGTCCGGCCCT-GTTCACGTTAT | 2.82E-1 |
| ipu-miR-1388 | BCAR1_3UTR | 8 | -15.40 | CATGGGAGCTTATAGAGACACTGAGAT  \|\| \|\|\| \|\|: \|\|\|\|\|\|\| AGTACC-CGA-CTGCTT--GGACTCTA | 1.3E-1 |
| ipu-miR-27a | BCAR1_3UTR | 9 | -15.90 | ATGGGAGCTTATAGAGACACTGAGAT  \|\|\|:\|\| \|\|\| \|\|\|\|\| \|\|  TCGCCTTGA--ATC--GGTGACACTT | 1.3E-1 |
| ipu-miR-460 | BCAR1_3UTR | 60 | -15.40 | TTATCC-CATGTCCATGCTTTGTG  :\|\|\|\| \|\|\| \|\|\|\| :\|\|\|\| CGTAGGTGTA-ACATACGCGACAC | 1.3E-1 |
| ipu-miR-122 | BCAR1_3UTR | 749 | -16.50 | GCTCACCTTTCTCCCACTCCG  \|\|\|\| \|\| \|\| \|\|\|\|\|\|: TTTGTGGTAACAGTGTGAGGT | 2.18E-1 |
| ipu-miR-20a | BCAR1_3UTR | 866 | -16.60 | GC-CAAAGCCGTTCTCCATTGCAGT :\| \|\|\| \|:\|\| \|\|:\|\|\|\|\|\| TGAAGTTC-ACGAG-TGTGACGTCA | 1.84E-1 |
| ipu-miR-217 | BCAR1_3UTR | 867 | -16.00 | CCAAAGCCGTTCTCCATTGCAGTG \|\|\|\| \| \|\|\|\|: \|\|\|\|\|\|: GGTT-AGTCAAGG-ACTACGTCAT | 1.84E-1 |
| ipu-miR-17b | BCAR1_3UTR | 868 | -20.40 | CAAAGCCGT-TCTCCATTGCAGT  \|\| :\|\|\|\|\|:\|\|\|\|\|\| CGAACTTCACGGAGGTGACGTCA | 1.84E-1 |
| ipu-miR-17a | BCAR1_3UTR | 869 | -20.80 | AAAGCCGT-TCTCCATTGCAGT  \|\| \|\| :\|\|\|\|\|:\|\|\|\|\|\| GATCTTCACGGAGGTGACGTCA | 1.84E-1 |
| ipu-miR-460 | BCAR1_3UTR | 873 | -16.90 | CCGTTCTCCATTG-CAGTGCTGTA  \|:\|:\| \|\|\|\|\| \|:\|\|\|\|\|  CGTAGG-TGTAACATACGCGACAC | 1.84E-1 |
| ipu-miR-101a | BCAR1_3UTR | 875 | -18.10 | GTTC-TCCATTGCAGTGCTGTA  \|\|\| \|\| \|\|\|\|:\|\|\|\|\| GAAGTCAATAGTGTCATGACAT | 1.84E-1 |
| ipu-miR-214 | BCAR1_3UTR | 882 | -20.10 | ATTGCAGTG-CTGTACCCTGCTGG  :\|\|\| \|\| \|\|\|\| \|\|\|\|\|\|\|  AGACG-GACAGACA-CGGACGACA | 1.84E-1 |
| ipu-miR-199a-2 | BCAR1_3UTR | 883 | -15.30 | TTGCAGTGCTGTACCCTGCTGG  \|\|:\|\| \|\|:\| \|\|:\|\|\|  TTGGTTAC-ACGTCTGATGACA | 1.84E-1 |
| ipu-miR-187 | BCAR1_3UTR | 885 | -15.20 | GCAGTGCTGTACCCTGCTGGACAGGG :\| \| \|\|\|\|:\| \| :\| :\|\|\|\| \|: TGAC-CGACGTTG--TGTTCTGTGCT | 1.84E-1 |
| ipu-miR-181a-1 | BCAR1_3UTR | 1169 | -15.50 | CTCACCTTTTGCG--AAATGTT \|\|\|\|\|\| \|\|\| \|\|\|\|\|\| GAGTGGCTGTCGCAACTTACAA | 3.72E-1 |
