# Supplementary material for: The Y chromosome sequence of the channel catfish suggests novel sex determination mechanisms in teleost fish
Source: BMC Biol. 2019 Jan 25;17:6. doi: 10.1186/s12915-019-0627-7 (PMC6346536; doi:10.1186/s12915-019-0627-7)
Supplement: Supplementary file 4 — Table S4. Differentially expressed genes in germ cell-Sertoli cell junction signaling pathway. (DOCX 14 kb) [file 12915_2019_627_MOESM4_ESM.docx]

**Table S4** Differentially expressed genes in germ cell-sertoli cell junction signaling pathway.

| Pathway name | | | p-value | |
| --- | --- | --- | --- | --- |
| Germ Cell-Sertoli Cell Junction Signaling | | | 6.98E-03 | |
| Symbol | Gene Name | Identifier | Location | Type(s) |
| BCAR1(p130CAS) | breast cancer anti-estrogen resistance 1 | NP_001128605.1 | Plasma Membrane | Enzyme |
| MAP3K10 | Mitogen-Activated Protein Kinase Kinase Kinase 10 | XP_689424.4 | Cytoplasm | Kinaze |
| MYO7A | Myosin VIIA | NP_694515.1 | Cytoplasm | Enzyme |
| PIK3C2A | Phosphoinositide 3-Kinase-C2-Alpha | XP_700029.4 | Cytoplasm | Kinase |
